# Cystic swelling and inflammation of MCL of knee joint managed with homeopathy; a case study with literature review and diagnostic pitfalls

**DOI:** 10.1016/j.jaim.2025.101182

**Published:** 2025-09-12

**Authors:** Shuvasree Bhattacharya, Pritam Goswami

**Affiliations:** a Consultant Homeopath and Independent Researcher, 29, Vidyasagar Sarani Ghosh Para Bally, Howrah, 711227, West Bengal, India; b Department of Pathology and Microbiology, Kharagpur Homeopathic Medical College and Hospital, India

**Keywords:** Cystic swelling of knee joint, Lysholm knee score and tegner activity scale, Ruta graveolens, Thuja occidentalis, Case report

## Abstract

Knee pain is the most frequently encountered complaint in clinical practice among diverse age groups. In India, osteoarthritis alone covers 25–35 % of all rheumatological conditions. That stands out as one of the common causes of misdiagnosis of all other soft tissue and ligamentous diseases of the knee joint. However, in light of common clinical manifestations like osteoarthritis, many cases go neglected *and remains in the* grey area of literature. Here we are reporting a case of a 65-year-old female patient who presented with knee pain, and difficulty walking, and she was previously diagnosed with osteoarthritis. However, ultrasonography (USG) revealed a cystic enlargement measuring 12.3 × 5.4mm distal to the medial-cutaneous ligament (MCL) with inflammation and local edema. Following a thorough case evaluation, the patient was prescribed Ruta graveolens 200CH, followed by Thuja occidentalis 200CH. The anti-inflammatory, anti-tumor, and anti-cancer properties of both these medicines have been thoroughly demonstrated via many scientific experiments. We believe the positive effect is due to their alkaloids, but we have not questioned how they maintain their qualities at such high dilutions. The Lysholm Knee Score and Tegner Activity Scale (TLSS) were used to quantify progressive improvement. Significant improvement was observed with the following remission of cystic edema and local irritation. The TLSS climbed to 78 at the end of treatment from 56 at the beginning. Despite being a rare clinical presentation of knee pain, this case report is valuable in homeopathy since it is the first attempt to analyze knee joint functionality using TLSS.

## 1 Introduction

The bone and musculoskeletal (MSK) systems dominates the domain of acute and chronic morbidities that require rapid patient care and management. MSK diseases cause disability and impose a significant personal, societal, and economic burden around the world, and adolescent pain is regularly described in epidemiological research, with the knee being the most typically afflicted site [1, 2]. Knee pain is a frequent clinical complaint in adults, affecting over 50 % of the population over 50 [3]. According to a recent study, the prevalence of osteoarthritis in India has increased from 23.46 million in 1990 to 62.35 million in 2019. The age-standardized prevalence of OA has risen from 4895 (95 % uncertainty interval) in 1990–5313 in 2019, per 100,000 individuals [4]. Females have a much higher prevalence, incidence, and DALYs from OA and knee OA than males. Another study found a 45.3 % prevalence rate of knee pain in adolescents over the past 12 months, which is congruent with a study conducted by Bhakti in India in 2021 when 43.3 % of adolescents reported knee discomfort [5]. Studies on knee pain prevalence among teenagers show varying results: A 2015 study in Brazil reported 22.6 %, while Finland's 1995 study found 18.5 %. In England (1984) and Denmark (2011), rates were higher at 30 % and 27 %, respectively [[6], [7], [8], [9]]. Another Danish study showed 28 %. Conversely, a Canadian study reported a lower prevalence of 7.4 % [10, 11]. These differences may reflect regional factors such as lifestyle, sports involvement, and healthcare access. In India, such studies have not been done so far but the gradual escalation in several orthopedic cases depicts the demands to explore the grey area of understanding.

**Fig. 1:**
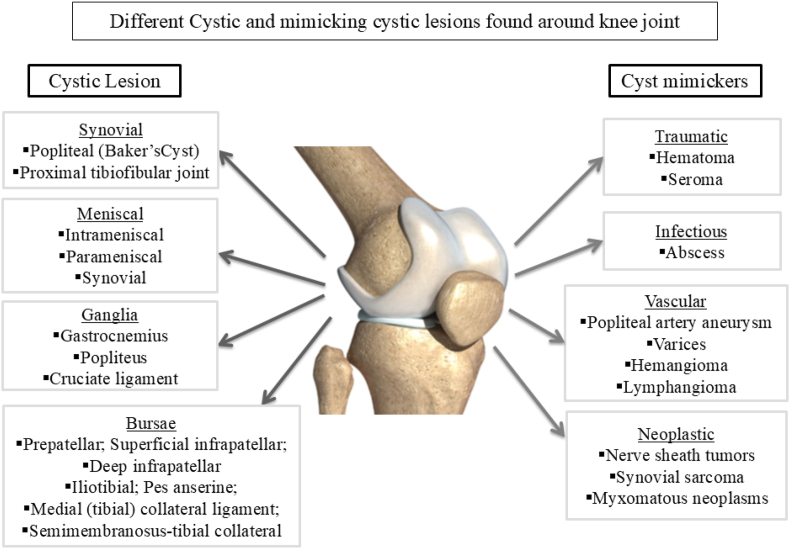
Showing different lesions around the knee joint that wither presents as Cystic swelling or mimics as cystic swelling which if not diagnosed properly may turn around to be a reason for unfavorable outcome in case of a pain in Knee joint.

The disparities in these percentages could be due to race, sample characteristics, and differences in awareness and reporting of knee pain symptoms. Additional research is needed to delve deeper into this topic. Several cystic pathologies have been observed around the knee joint, as well as soft tissue lesions (Fig. 1) in the surrounding structures, which are usually presented as knee discomfort in clinical practice. That is why it is critical to conduct a thorough history in conjunction with diagnostic tests to provide appropriate treatment. The increased frequency and consistency in the clinical presentation of knee pain frequently leads to misdiagnosis as knee OA in day-to-day clinical practice [12]. The financial impact of MSK diseases on the healthcare system is significant, since one in every six people with knee discomfort seeks medical attention each year, and one-third of them become disabled [13]. Epidemiological studies have consistently demonstrated that teenagers suffer from self-reported pain, with pain in the knee joint being one of the most common sources of suffering. Musculoskeletal issues, such as knee pain, impose a significant burden on individuals, society, and the economy worldwide [14]. As a result, it is critical to understand adolescents’ knee discomfort, including its causes and risk factors. In India, no comparable research has been conducted to elucidate and treat the underlying causes of misdiagnosis. However, even though the European League Against Rheumatism (EULAR) and the American College of Rheumatology have established unambiguous diagnostic criteria [Table-1] [15, 16], knee joint aches are frequently identified as OA. In this case study, we intend to present a novel case report on a cystic lesion of the knee joint that was previously misdiagnosed as OA. This case was managed with homeopathic interventions along with physiotherapy.

**Table 1:** Showing EULAR and ACR criteria for diagnosis of Osteoarthritis of the Knee joint.

European League Against Rheumatism (EULAR)	American College of Rheumatology (ACR)
Pain on the Knee joint Age more than 40 years, Movement-related knee pain, Morning stiffness less than 30 minutes, Association of functional limitation, Have one or more typical examination findings (Crepitus, restricted movement, and bony enlargement). Along with Relevant clinical History	Pain in the Knee Joint Over 50 Years of Age Less than 30 minutes of morning stiffness Crepitus on active motion Bony tenderness Bony Enlargement No palpable warmth on Synovium Along with +ve Radiological findings and Relevant Clinical History

## 2 Case presentation

Mrs. N.G., a female patient around 65 years old, came to the clinic complaining of pain in her left knee joint for the past 6 months. On further enquiry the patient narrated about the pain was localized in nature in the back of the knee joint and having a splinter like sensation. The patient was unable to walk normally as she was having a splinter like sensation because of which she was unable to extend left leg properly. The discomfort was relieved when sitting or stretching the legs, or when hot bags were applied, but it exacerbated when walking, folding the legs, or changing positions. Despite being treated by an orthopedic, the patient was disappointed with the results, prompting her to seek an alternative. Clinical history revealed that the patient has hypertension and is taking anti-hypertensive medications. Past history shows that Cholecystectomy was performed because to calculus cholecystitis. Following a thorough case history, the patient revealed no substantial history of trauma involving the component. Her general history revealed a low appetite with a preference for fish and acidic foods; thirst was moderate, with a consumption of 1.5–2lit/day and a dry white coated tongue. There were no particular complaints about urination; however the patient did report a long history of constipation with no history of bleeding per anus. Sleep was allegedly disturbed due to pain throughout the phases of new and full moon. Patient has complained about being intolerant to chilly weather, which exacerbates all of her ailments. The patient was irritable due to her suffering. Patient also reported to be dominant in nature and have fixed ideas as her son described.

## 3 Clinical examination

Her blood pressure was 132/84 mm/Hg, and she weighed 76 kg. During Systemic Examination no atrophy was noted in both lower limbs. On palpation, joint crackling was detected in the knee joint, but no effusion edema was found around the joint. No palpable edema was found on the anterior or posterior surface of the left knee joint. No lesion or injury was discovered during the evaluation of the component. The lower limb bones' curvature was maintained, and the patient's limping stride was noted because she was unable to stretch her left foot due to pain.

## 4 Diagnostic assessment

**Fig. 2:**
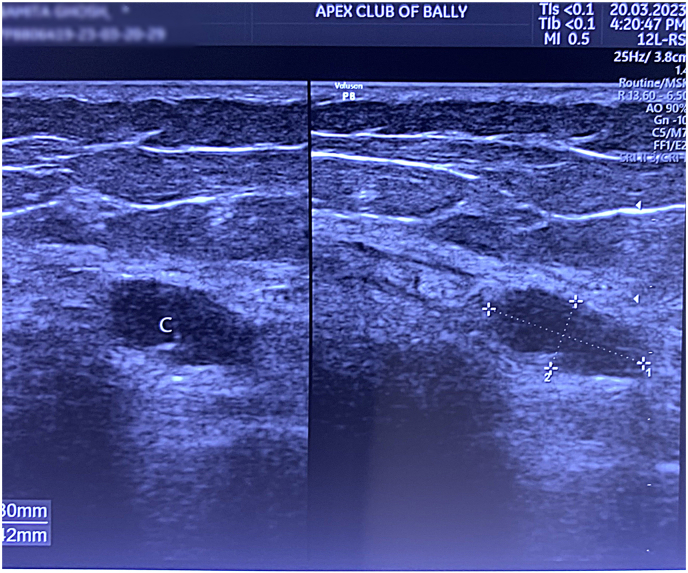
Showing Cystic swelling (Arrow) at the base of distal MCL measuring 12.3 × 5.4 mm with swelling and edema.

The patient was advised to go for Ultrasonography (USG) of her left knee joint in order to exclude association of any other soft tissue pathology around the knee joint as the presenting complaint for such condition are also localized pain knee joint. The USG revealed presence of a cystic swelling (12.3 × 5.4mm) (Fig. 2) at the distal MCL along with swelling and edema. Table 2: Contains all the laboratory parameters assessed during the beginning of treatment (see Table 3).

**Table 2:** Showing laboratory investigations at baseline

**Hb%**	11.9 gm%
**RBC**	4.3 million/cmm
**TLC**	5800/cmm
**ESR**	65 mm
**FBS**	105 gm/dl
**PPBS**	126 gm/dl
**Serum Calcium**	9.5 mg/dl
**25 (OH) Vit-D3**	19.35 ng/ml

**Table 3:** Time line of the treatment with successive follow ups.

Date of Visit	Presenting Symptoms	Prescribed Medicine
13/03/2023	Pain and difficulty in movement in left knee joint. Occasional association of splinter like sensation. Pain is better from hot application, in motion, in rest.	Rhus toxicodendron 30CH/4D followed by Placebo
21/5/2023	Pain has not improved, USG revealed the presence of Cystic swelling at the base of distal MCL. Others symptoms were unchanged.	Ruta 200CH/4Doses followed by Placebo for 2 months. Tab Vitamin D3 1 Tab x 60 Days
17/7/2023	Intensity of the localized pain and splinter like sensation was reduced but the difficulty in walking was unchanged.	Ruta 200/2Doses followed by Placebo for 2 months
11/9/2023	Localized pain was better splinter like sensation was absent. But pain typically increased during the time of new moon and full moon. Considering the mental symptoms, Prescription was changed.	Thuja 200CH/2D followed by Placebo for 2 months.
16/11/2023	Pain was diminished along with much improvement in the stiffness was noted. Same medicine was repeated and the patient was instructed to go for further USG evaluation of Left knee-joint.	Thuja 200CH/1D followed by Placebo for 1 month
23/12/2023	Pain and stiffness was reduced but difficulty in walking persisted. USG revealed normal appearance of the soft tissue around left knee joint.	No further Medicine was Prescribed. Advised to continue physiotherapy for alleviation of local discomfort.

## 5 Functional assessment

As the patient was being conventional painkillers so we initiated the treatment with a palliative approach along with preventive management where the patient was advised to use knee brace in order to reduce the pain while walking or standing. After 7 days the patient came up with USG report where it revealed presence of Cystic swelling, that helped us to understand the reason for pain in the back of the knee which was getting triggered every time during complete flexion or extension of the leg. The functional ability and nature were evaluated to understand performance and activity restrictions with the help of Lysholm Score and Tegner Activity Scoring (TLSS).

## 6 Therapeutic intervention

The therapeutic intervention was primarily directed to manage the pain and improvement of quality of life. As we know that cystic swelling is a surgical intervention so it was challenging to approach as if increase in the size of the cystic swelling may lead to further deleterious complications.

### 6.1 Follow up

**Fig. 3:**
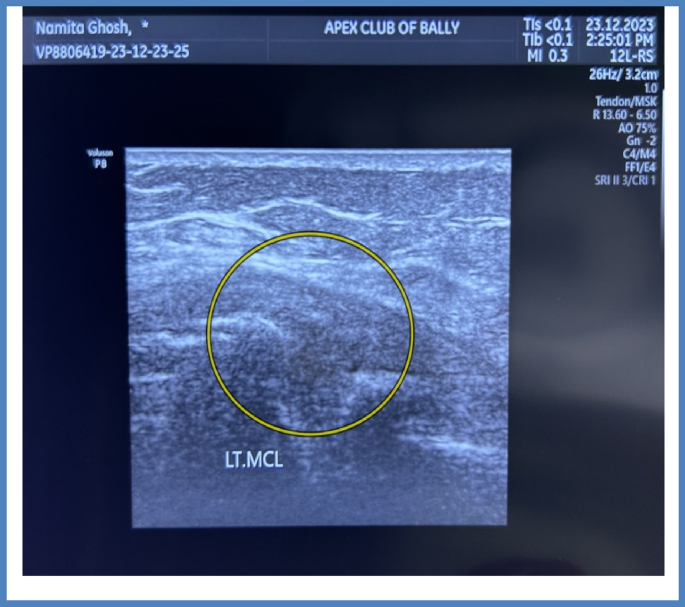
Showing complete resolution of the cystic selling (Circle) with no evidence of local soft tissue edema and swelling after intervention.

**Graph 1:**
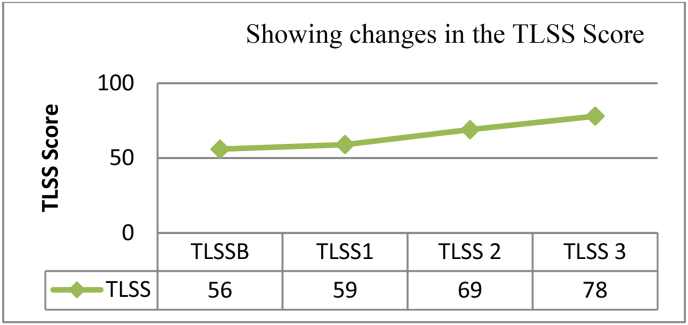
Showing gradual improvement at the TLSS during the period of treatment. Here TLSSB stands for score at Base-line; TLSS1, TLSS2 and TLSS3 stand for scores at successive follow ups.

Considering the patient's symptoms the patient was prescribed Rhus toxicodedron 30/4doses (4D) doses in order to alleviate the localized pain. After one week when the patient reported with USG reports then she was prescribed with Ruta 200CH/4doses (4D) followed by placebo for 28 days. After two months, the patient's knee pain and gait improved, therefore the medication was repeated. This time, Ruta 200CH/2 dosages (2D) were prescribed to prevent any adverse reactions to the drug. As the patient's condition improved, Thuja 200CH/2 dosages (2D) were administered to supplement Ruta's activity, taking into accounts all of the symptoms after two months. After two months, Thuja 200CH/1dose (D) was administered again, and the patient was sent for another evaluation, which showed that the cystic edema had subsided and that the soft tissue surrounding the left knee joint was normal (Fig. 3). A detailed timeline of the treatment has been given in the Table-2. Along with improvement in the symptoms we observed subsequent improvement in the TLSS score. TLSS at the base line was (TLSSB- 56) which was increased to 59 and 69 during first (TLSS1) and second TLSS (2) follow-up. At the end of the treatment TLSS were 78 with co-ordinated with marked improvement of the symptoms. A graphical representation of the change in TLSSS score has been given in Graph-1.

### 6.2 Adverse events

No adverse event was administered during the period of treatment.

### 6.3 Homeopathic aggravation

During intervention no occurrence were encountered following administration of homeopathic drugs.

### 6.4 Possible causal attribution

Table 4 provides the MONARCH criterion [17]. A score of 9 on the MONARCH criteria indicates that homeopathy was causally attributed to the individual's asserted improvement in the condition. The principal symptoms were scored on whether they improved after taking the medication and whether the outcomes were noticeable within a reasonable time limit. The treatment resulted in an improvement in general well-being. Changes observed in imaging studies (USG) corroborated the findings. Examining potential reasons of the illness that go beyond medication is a crucial component of the criterion.

**Table 4:** Showing the possible causal attribution by MONARCH score.

MONARCH Inventory			
Items	Yes	No	Not Sure
1. Was there an improvement in the main symptom or condition, for which the homeopathic medicine was prescribed?	+2		
2. Did the clinical improvement occur within a plausible time frame relative to the drug intake?	+1		
3. Was there a homoeopathic aggravation of symptom? (need to define in glossary)		0	
4. Did the effect encompass more than the main symptom or condition, i.e., were other symptoms, not related to the main presenting complaint, improved or changed)?		0	
5. Did overall well-being improve? (suggest using a validated scale or mention about changes in physical, emotional and behavioral elements)	+1		
6: (a) Direction of cure: Did some symptoms improve in the opposite order of the development of symptoms of the disease?			+1
(b) Direction of cure: Did at least one of the following aspects apply to the order of improvement of symptoms: • From organs of more importance to those of less importance? • From deeper to more superficial aspects of the individual? • From the top downward?		0	
7. Did “old symptoms” (defined as non-seasonal and non-cyclical symptoms that were previously thought to have resolved) reappear temporarily during the course of improvement?		0	
8. Are there alternate causes (other than the medicine) that – with a high probability – could have caused the improvement? (consider known course of disease, other forms of treatment and other clinically relevant interventions)		+1	
9. Was the health improvement confirmed by any objective evidence? (e.g., investigations, clinical examination, etc.)	+2		
10. Did repeat dosing, if conducted, create similar clinical improvement?	+1		
Total score- 9			

## 7 Discussion

Knee discomfort and dysfunction can be caused by soft tissue inflammation and adhesions, which are often overlooked. Abnormalities in the fat pad, synovial plica, supra-patellar pouch, and lateral retinaculum can arise independently or in conjunction with significant ligamentous, chondral, or meniscal injuries [18]. Under normal physiological settings, these entities do not influence knee biology or biomechanics. However, without proper diagnosis and treatment, these structures can cause substantial discomfort and hinder joint homeostasis, leading to poor patient outcomes. According to a concerning study carried out in the Republic of China, when beneficiaries (N = 127,570) sought treatment for knee discomfort, the majority (99.7 %) were originally given a non-specific knee diagnosis; however, this only happened in 16.5 % of the instances (n = 20,042) after the two-year follow-up [19]. Access to proper treatment is frequently restricted in low- and middle-income countries like India and is reliant on a patient's financial capacity, insurance, availability of various treatment alternatives, and accessibility to healthcare services [20]. In addition, doctors in the government healthcare system had to handle the symptoms because the bulk of their patients were from rural India, and their low socioeconomic level and lack of health insurance further contributed to this. To better understand different treatment methods and approaches for knee pain, another multi-centric KAP study was carried out. The results showed a substantial gap, with conservative treatment being the preferred option over interventional procedures [21]. This evidence-based case report highlights the importance of diagnosing and treating these non-ligamentous soft tissue anomalies, highlighting the range of homeopathy, in contrast to the ongoing trend.

Based on the symptoms, two medicines were prescribed: Thuja occidentalis 200CH and Ruta graveolens 200CH potency. According to evidence that was mentioned in the writings of Hippocrates, Dioscorides, and Pliny, Ruta has been utilized in phytomedicine for centuries [22]. These substances have strong analgesic, anti-inflammatory, and anti-cancer actions and considerable metabolic effects such as xanthine oxidase inhibitory, anti-hyperglycemic, and anti-hyperlipidemic effects [23]. In addition to inducing DNA damage pathways and cell migration, Ruta graveolens at a 200CH dilution has been shown to have a strong anti-tumor impact [[24], [25], [26]]. Apart from these Rutin, which is an important alkaloid of the Ruta has been proven to prevent the cascade of neuronal degeneration by blocking the activity of monoamine oxidase, acetyl-cholinesterase [27]. In addition to Ruta graveolens, Thuja occidentalis, another medication that was utilized in this instance, also has strong positive effects at ultra-dilution. Thuja in homeopathic preparation has been shown in studies to effectively induce apoptosis in functional p53-expressing mammary epithelial carcinoma cells and to abrogate intracellular reactive oxygen species (ROS). Additionally, it has been shown to prevent p53-activation, knock down p53, or inhibit its functional activity, which significantly reduces the generation of ROS [28]. In addition, extracts from Thuja occidentalis have strong anti-cancer properties and can stop cell proliferation and angiogenesis [29]. The evidence supports the justification for using the medications with positive results.

Homeopathic remedies prescribed in modest doses spaced intermittently throughout time are supposed to serve as signals from the body that stimulate the organism's allostatic biological responses to stress network, resulting in nonlinear modulatory and self-organizing change. Potential mechanisms include time-dependent sensitization (TDS), a type of adaptive plasticity/metaplasticity characterized by gradual amplification of host responses that reverse direction and fluctuate at physiological limits [30]. We believe that the initial favourable effect in alleviating localized pain followed by remission of cystic swelling is related to the action of these two homeopathic medicines.

## 8 Conclusion

The diagnosis of these lesser-known causes of knee pain and dysfunction necessitates a thorough awareness of the anatomy and pathophysiology of these tissues, as well as a high level of clinical suspicion. Treating these pathologic structures with surgery for other major knee pathologies (e.g., meniscal, ligamentous, chondral) may result in less intra-articular discomfort, better patient outcomes, and joint homeostasis. From a homeopathic standpoint, this case report has is important because it is possibly the first attempt to record a relatively unknown source of knee pain from both diagnostic and therapeutic perspectives.

## Patient consent

Formal patient consent has been taken.

## Patients perspective

Initially the patient was diagnosed with OA but she was not improving following administration of medicines, then she shifted to homeopathy with little hope. As per the patient “Initially I thought I will not be able to walk like I did, but now I am satisfied with the result as I can perform my daily house-hold activities. I am satisfied with the outcome and wish to consult homoeopathy in future if needed.”

## Author's contribution

PG: Diagnosis and Treatment and Prepared the Manuscript; SB: Helped in the treatment and follow up along with data curation and formal analysis.

## Declaration of generative AI in scientific writing

We hereby declare that no Artificial Intelligence (AI) assisted technical tools or Image creators were used in the preparation of this manuscript.

## Funding sources

Not Applicable.

## Declaration of competing interest

Authors declare none.
